# The Healthy Body Image Intervention and Reduction in Eating Disorder Symptomatology and Muscle Building Supplement Use in High School Students: A Study of Mediating Factors

**DOI:** 10.3389/fpsyg.2022.803654

**Published:** 2022-06-28

**Authors:** Kethe Marie Engen Svantorp-Tveiten, Andreas Ivarsson, Monica Klungland Torstveit, Christine Sundgot-Borgen, Therese Fostervold Mathisen, Solfrid Bratland-Sanda, Jan Harald Rosenvinge, Oddgeir Friborg, Gunn Pettersen, Jorunn Sundgot-Borgen

**Affiliations:** ^1^Department of Sports Medicine, Norwegian School of Sport Sciences, Oslo, Norway; ^2^Center of Research on Welfare, Health and Sport, Halmstad University, Halmstad, Sweden; ^3^Department of Sport Science and Physical Education, University of Agder, Kristiansand, Norway; ^4^Faculty of Health, Welfare and Organization, Østfold University College, Fredrikstad, Norway; ^5^Department of Sports, Physical Education and Outdoor Studies, University of South-Eastern Norway, Kongsberg, Norway; ^6^Department of Psychology, UiT – The Arctic University of Norway, Tromsø, Norway; ^7^Department of Health and Care Sciences, UiT – The Arctic University of Norway, Tromsø, Norway

**Keywords:** body image (MeSH), eating disorder (ED), mental health, adolescence, prevention, muscle building supplements

## Abstract

**Background:**

Mediation analysis is important to test the theoretical framework underpinning an intervention. We therefore aimed to investigate if the healthy body image (HBI) intervention’s effect on eating disorder (ED) symptomatology and use of muscle building supplements was mediated by the change in risk and protective factors for ED development and muscle building supplement use.

**Methods:**

This study used data from the HBI intervention: a cluster randomized controlled universal intervention aiming to promote positive body image and embodiment and reduce the risk for ED development including 30 schools in Norway. A total of 1,713 (37% boys) participants were included in the analyses. Conditional latent growth curve analyses were performed to test for indirect effects on ED symptomatology and weekly frequency of protein and creatine supplement use measured at the 12-month follow-up *via* change in the proposed mediators.

**Results:**

In girls, the reduction in ED symptomatology was mediated by positive changes in protective factors (self-esteem and body image flexibility) and reductions in risk factor scores (perceived media pressure and thin appearance internalization). Comparable changes in protective and risk factors among boys played no mediating role.

**Conclusion:**

Interventions aiming to reduce the risk of ED development in girls may benefit from aiming to enhance self-esteem and body image flexibility and reduce perceived media pressure and thin appearance internalization. Future studies should investigate the casual relationship between muscle building supplement use and risk and protective factors for ED development in both girls and boys.

## Introduction

Evidence suggests that interventions aiming to prevent eating disorders (EDs) should target established risk and protective factors for ED development such as internalization of appearance ideals, perceived appearance pressure, self-esteem, and peer environment and positive emotion coping strategies, such as body image flexibility (Watson et al., 2016; Le et al., 2017; Rogers et al., 2018; Stice and van Ryzin, 2019; Linardon et al., 2021). The reviews also highlight that targeting media literacy using interactive education, challenging cognitive dissonance with multiple sessions, seems to be effective in universal ED prevention in adolescents (Stice et al., 2008, 2013; Yager et al., 2013; Le et al., 2017).

Sociocultural theoretical framework focus on the role of social and cultural influences on individual development (Lantolf, 2000). This framework is often used to describe and understand how social and cultural factors influence ED development. Sociocultural frameworks has the past decade also been used to explain muscle-oriented body image and muscle building behaviors (Rodgers et al., 2012, 2020; Girard et al., 2018a,b; Carvalho and Ferreira, 2020). The sociocultural theories of body image, ED symptomatology, and muscle building behaviors have been tested and modeled in cross sectional and longitudinal studies (Rodgers et al., 2011, 2012, 2014, 2015; Girard et al., 2018a,b; Carvalho and Ferreira, 2020; Burke et al., 2021; Maher et al., 2021; Schaefer et al., 2021). In summary, these studies suggest that perceived appearance pressure from sociocultural sources (e.g., media, family, and peers) explains body image, ED symptomatology, and muscle building behaviors through mechanisms of appearance internalization and social comparisons. Moreover, recent studies have suggested social media as a source of appearance pressure in explaining body dissatisfaction, ED symptomatology, and muscle building behaviors (Rodgers et al., 2020; Jarman et al., 2021).

Further, the recent evaluations of sociocultural models of body image and ED symptomatology have also included the role of protective factors within the field of positive body image (Tylka and Wood-Barcalow, 2015), such as self-esteem, self-compassion, and body image flexibility. These models highlight that self-esteem may act as a protective factor against appearance pressure, internalization, and comparison (Rodgers et al., 2014, 2020), which suggests that individuals with higher self-esteem may experience less appearance pressure, internalization of unrealistic appearance standards, and more likely to resist comparing themselves with others. However, only one study, and among girls only, has included self-esteem as a potential mediator for intervention effect (Agam-Bitton et al., 2018). Moreover, self-compassion and body image flexibility act as moderators between appearance internalization and social comparison and ED symptomatology (Perey and Koenigstorfer, 2020; Maher et al., 2021). This indicates that the risk of ED development caused by appearance internalization and social comparison may be outnumbered by body image flexibility and self-compassion. Body image flexibility is a core component of positive body image and reflects the ability to maintain behaviors that are consistent with chosen values when negative feelings, thoughts, body sensations, or memories related to one’s own appearance are experienced (Sandoz et al., 2013). High levels of body image flexibility is found to be inversely associated with less adaptive emotion coping strategies, disordered eating (DE), and negative body image and positively linked to positive emotion regulation strategies, body satisfaction, self-compassion, well-being, and quality of life (Rogers et al., 2018). Importantly, there is evidence that body image flexibility could be a component in the ability to hold a positive body image while experiencing body dissatisfaction (Webb, 2015).

In contrast to dieting or weight reduction behaviors, muscle building behaviors have been given less attention in ED prevention interventions (Doley et al., 2021). Research suggests that the use of muscle building supplements, such as protein and creatine supplements, correlates with negative body image and positively with muscular-oriented body dissatisfaction and use of muscle building behaviors in adolescents (Yager and O’Dea, 2014; Yager and McLean, 2020). Muscle building supplement use has also been found to be explained by core ED symptoms, shape concerns, and restrictive eating in young adult males and females; binge eating and compulsive exercise in males; and bulimic behaviors in females (Nagata et al., 2020). In addition, engagement in muscle enhancing behaviors, which include supplement use, predicts dietary restraint in both boys and girls (Rodgers et al., 2020). Yet, a recent study indicates that protein and creatine supplement use could be considered a symptom of DE in boys, but not in girls (Svantorp-Tveiten et al., 2021a). Therefore, to better capture a broader range of factors related to DE and EDs, especially in boys, it seems reasonable to include muscle building supplement use within an intervention aiming to prevent DE and EDs in a sample also including boys. It may be reasonable to assume that interventions with similar methodological strategies as successful ED prevention programs could be effective in preventing muscle building supplement use, as muscle building behaviors may be explained by similar psychological concepts as DE and ED (Rodgers et al., 2020). In addition, the media literacy approach is found to be successful in preventing a wide range of unhealthy behaviors and attitudes other than DE and EDs (Jeong et al., 2012; Rodgers et al., 2020).

Few studies have tested if change in the suggested and targeted risk factors is the actual cause for the intervention’s effect on ED symptomatology or similar main outcomes in universal ED prevention. The perceived media pressure is normally targeted in ED prevention programs along with internalization of a general media or thin appearance ideal (Schwartz et al., 2019). However, the perceived media appearance pressure has not been evaluated as a potential mediator for intervention effects, and only three studies have investigated general media internalization as a mediator in universal prevention interventions (Gumz et al., 2017; Wade et al., 2017; Agam-Bitton et al., 2018). The previous studies have assessed general media internalization and to the best of our knowledge, no studies have investigated actual thin appearance internalization as a mediator in universal ED prevention programs. In addition, athletic and muscular appearance internalization has not been suggested as a mediator in previous studies, which may be important in studies including boys and girls who identifies within the muscular-oriented dimension of body image. Further, no study has included protective factors for ED symptomatology, beyond self-esteem, as mediators in universal ED prevention. Importantly, no studies have considered these mediators in relation to muscle building supplement use and within a randomized controlled trial design allowing to draw casual conclusions. Additionally, investigating if mediation and mechanisms of change from ED prevention intervention differ between girls and boys has been requested by others (Wade et al., 2017) and could provide to the understanding of current evidence that suggests that boys tend to benefit less from universal ED prevention programs than girls (Le et al., 2017). Knowledge about mediating constructs in interventions is important to test the theoretical foundation that the intervention builds on (Stice et al., 2013), to increase the understanding of the causality among variables (Jose, 2016), and to determine which risk and protective factors may have been most important to target in the current intervention in order to further develop or fine-tune the intervention.

The healthy body image (HBI) intervention (Sundgot-Borgen et al., 2018) is a universal school-based intervention aiming to promote positive body image and embodiment and reduce the risk of ED development through targeting risk and protective factors within the sociocultural theory and positive body image (Rodgers et al., 2014, 2020; Piran, 2015; Levine and Smolak, 2016). The HBI intervention targets body image, media literacy, and lifestyle, and it is multicomponent in nature. It uses an interactive delivery strategy aiming to reduce risk for ED development through the promotion of students’ critical understanding of the influence of social and mass media and peers (Sundgot-Borgen et al., 2018). The HBI intervention has shown to be effective in reducing several risk factors for and enhancing protective factors against ED development in adolescent girls and boys (Svantorp-Tveiten et al., 2021b), muscle building supplement use in adolescent boys (Svantorp-Tveiten et al., 2021b), to positively affect eating habits and sleep duration (Sundgot-Borgen et al., 2020a), and to strengthen positive body image in girls (Sundgot-Borgen et al., 2019) through increased self-esteem (Sundgot-Borgen et al., 2020b). However, the effect of the HBI intervention was weaker among boys compared to girls with respect to psychological outcomes. This present study extends the current knowledge about ED symptomatology and muscle building supplement use as outcomes following participation in the HBI intervention. Additionally, the present study brings ahead new knowledge about the mediating role of a broader set of risk and protective factors that may explain why the intervention works. Formally, we examined if the previously reported reduced ED symptomatology observed in girls and muscle building supplement use observed in boys (Svantorp-Tveiten et al., 2021b) were mediated by the change in self-esteem, body image flexibility, thin appearance internalization, athletic and muscular appearance internalization, and perceived media pressure. We also aimed to explore if the change in the proposed mechanisms were similar in girls and boys. We hypothesized that the intervention effect on ED symptomatology and muscle building supplement use was mediated by the change in thin appearance internalization, athletic and muscular internalization, perceived media pressure, self-esteem, and body image flexibility in boys and girls.

## Materials and Methods

### Participants and Procedure

In the fall of 2016, all public and private high schools in the Oslo and Akershus counties in Norway were asked to take part in the study. The request and information about the study were presented to the school principals and administrators. Of the 50 eligible schools, 30 agreed to participate. A total of 3,947 students, aged 16–18 years, and attending the 12th grade were eligible for inclusion. We arranged a meeting at each school and all students were thoroughly informed about the study aims and the implication of participation. They were also requested to provide their email addresses to be reachable. The students subsequently received an information letter, an invitation to take part in the study, and an informed consent letter together with the questionnaires.

After baseline testing, all the students who completed the posttest questionnaire were offered a movie ticket (valued at $15). Due to limited funding, we were only able to invite participants who had consented and partially answered the baseline questionnaire (T1) to the posttest (T2). Additional funding made it possible to re-invite all those who had consented at T1 for the 3-month follow-up (T3), thereby increasing the number of students participating from T2 to T3. All consenting students at T1 were re-invited to the 12-month follow-up (T4). The T2 questionnaire was sent out to the students within 7 days after they had participated in the last workshop.

**FIGURE 1:**
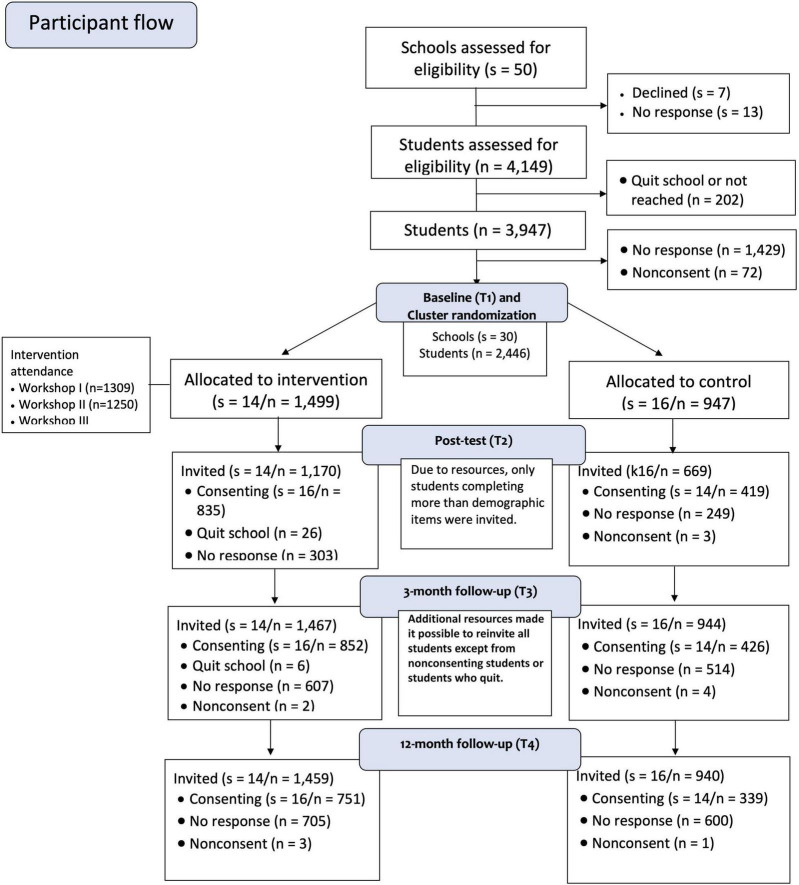
Recruitment, randomization, and participant flow. *s* = number of schools (clusters), *n* = number of students. The figure presents the number of consenting students. The number of students for the outcome measures varies between the different instruments.

For the current study, a total of 1,713 adolescents (intervention group: *n* = 1,130, 37% boys, control group: *n* = 583, 37% boys) responded on all included questionnaires at baseline and were included in the analyses. All questionnaires were completed electronically outside school hours using the web-based survey tool Survey Xact, by Ramboll, Norway. Figure 1 shows the number of consenting schools and participants at all measurement points for the main intervention study.

### Randomization

The schools (s) were randomly allocated to either the intervention (*s* = 14) or control group (*s* = 16) to equalize school size and to capture the urban-rural dimension, ensuring that all regions in the catchment area were included. To minimize contamination biases within the schools, a 1:1 ratio for cluster randomization was applied by an external professional unaffiliated with the project team. Each school represented an individual cluster to reduce the diffusion of effects due to information crossover between intervention and control students.

### The Norwegian Healthy Body Image Intervention

The aim of the intervention was to promote positive body image and embodiment and reduce the risk for ED development. The intervention involved three classroom-based and interactive 90-min workshops, focusing on the three topics; body image and self-esteem, social media, and lifestyle. The workshops were led by two female researchers specializing in body image, mental health, and sports and exercise science. Workshop attendance was recorded for every workshop by the intervention facilitators. The control schools had classes as usual and were informed that they could receive an abbreviated workshop after the 12-month follow-up. A comprehensive outline of the HBI intervention rationale, procedure, and content are described in a protocol paper (Sundgot-Borgen et al., 2018), and a brief summary of the intervention is given in Table 1.

**TABLE 1:** Overview of the content and aims of the three workshops in the HBI intervention.

#1 Body image	
**Main content:**	**Aim:**
Project introduction	Experience of meaningfulness and motivation
Influencing factors on body perception. What promotes and reduces positive body image, and how can we enforce the health promoting factors?	Body image and body acceptance
Where does body idealization come from? Why does it conflict with positive body image, and potential health consequences from striving for the idealized body?	Reduce idealization and internalization of body ideals
Fat talk and focus on lifestyle only related to appearance in everyday communication. To what degree do we participate, how does it make us feel, and can we reduce it?	Reduce fat talk and negative body talk
	Improve peer environment
Introduction to self-talk and self-esteem in WS#2	Stimulate motivation for next workshop

**#2 Social media**	

**Main content:**	**Aim:**

Social media perception and use. Empower yourself to choose mood enhancing over mood destructive content	Enhance media literacy
Extreme exposure without filter equals need to be critical to sources of information and awareness of retouching	Enhance media literacy
The nature of comparison, how to recognize destructive comparison and reduce its presence in everyday life	Reduce amount of comparison
Strengthen acceptance and love for individual differences, defining characteristics of ones’ own and among friends. Students tell and write down compliments to a friend and him/herself unrelated to appearance.	Improve positive self-talk
	Improve self-compassion
	Improve peer environment
Experiences and benefits of positive self-talk	Improve skills to strengthen self-esteem

**#3 Lifestyle**	

**Main content:**	**Aim:**

Benefits on body experience from listening to bodily needs such as physical activity and healthy eating	Improve experience of embodiment and positive body image
Truths and myth about lifestyle products and literature	Improve ability to reject exercise and nutritional myths—health information literacy
From aesthetic to functional focus; how can change in focus improve body experience and healthy lifestyle that again benefit well-being?	Change from potential unhealthy focus to healthy focus on the body
How may regular exercise and smart nutrition promote positive body image and what are the basic recommendations?	Body experience enhancing attitudes and behaviors

*Retrieved and adapted from Sundgot-Borgen et al. (2018).*

### Outcomes

#### Main Outcome Variables

The *ED symptomatology* was the main outcome variable and was measured with an empirically derived (Friborg et al., 2013) brief version of the eating disorder examination questionnaire (Fairburn and Beglin, 2008) consisting of 11 items (EDEQ-11). The items are scored on a seven-point Likert scale ranging from “no days”/“not at all” to “every day”/“Markedly” (e.g., *“Have you had a definite desire to have a totally flat stomach?”* or *“Has your shape influenced how you think about (judge) yourself as a person?”*). All items were added, and an average score was computed. The abbreviated version was found to correspond well with the complete 22-item instrument (Friborg et al., 2013) that discriminates well between patients with ED and healthy individuals (Rø et al., 2015). The internal consistency (α) was 0.94 and 0.91 in girls and boys, respectively.

The muscle building supplement use included protein and creatine supplement use. The protein and creatine supplement use was measured with self-developed questions on the use of supplements and on how many times per week the supplementation was consumed [e.g., *“How often during a regular week do you consume protein supplements* (e.g., *powders/shakes*)?”] with values ranging on a five-point Likert scale scored: 0 (never), 1.5 (1–2 days), 3.5 (3–4 days), 5.5 (5–6 days), and 7 (every day).

#### Mediators

*The Rosenberg self-esteem scale* (Rosenberg, 1965) is a 10-item instrument measuring global self-esteem using both negative and positive worded items that are scored on a four-point Likert scale ranging from strongly agree to strongly disagree (e.g., “*I feel that I’m a person of worth, at least on an equal plane with others”*). The total score ranged from 0 to 30 and negatively worded items were reversed so that a higher score represents a higher level of global self-esteem. The α-values were 0.90 and 0.92 for boys and girls, respectively.

*The body image acceptance and action questionnaire* (BIAAQ) (Sandoz et al., 2013) is a measure of body image flexibility and of the ability to openly experience and accept challenging emotions and thoughts and maintain behaviors that are consistent with chosen values (e.g., not engage in DE behaviors) when negative feelings, thoughts, body sensations, or memories related to one’s own appearance are experienced. The scale includes 12 items (e.g., *“I shut down when I feel bad about my body shape or weight”*) that are scored on a seven-point Likert scale ranging from “never true” to “always true.” The score ranges from 12 to 84 where negatively worded items were reversed for a higher score to reflect more body image flexibility. The α-values were 0.92 in girls and 0.85 in boys.

*The sociocultural attitudes toward appearance questionnaire-4* (SATAQ-4) (Schaefer et al., 2015) was developed to assess the societal and interpersonal aspects of appearance ideals. The questionnaire consists of five individual subscales scored on a five-point Likert scale ranging from “strongly disagree” to “strongly agree.” The subscales “thinness/low body fat internalization” (SATAQ-4 Thin), “athletic and muscular internalization” (SATAQ-4 Muscular), and “perceived pressure from media” (SATAQ-4 Media) were used. The subscale scores range from 1 to 5 where higher scores indicate a higher degree of internalization or perceived pressure (e.g., *“I want my body to look very lean,”* “*I spend a lot of time doing things to look more muscular,”* and *“I feel pressure from the media to improve my appearance”*). The α-values ranged from 0.85 to 0.94 for boys and from 0.91 to 0.95 for girls.

### Data Analyses

Independent sample *t*-test or Mann–Whitney *U*-test was performed to test if baseline characteristics differed between responders (responded at T1 and one follow-up measure) and dropouts (responded at T1 only). A *p*-value < 0.05 was considered to indicate a statistically significant differences between dropouts and responders.

To test if there were indirect effects from the intervention to the outcomes, measured at T4, through change in the proposed mediators (T1-T4), conditional latent growth curve (LGC) analyses were performed in Mplus 8.4 (Muthén and Muthén, 2017). All models were performed with the robust maximum likelihood estimator (MLR). Separate models were estimated for each of the proposed mediators, and for both boys and girls. In the conditional models, group belonging (intervention, control) was included as an independent variable, EDEQ, weekly frequency of protein supplement use, and creatine supplement use were included as dependent variables, together with the four waves of the proposed mediators. In all models, the intervention group was coded as 0 and the control group was coded as 1. Within all analyses, we controlled for the baseline scores of all dependent variables (Vickers and Altman, 2001).

To evaluate model fit, we used a combination of the following fit indices (Marsh, 2007); comparative fit index (CFI; > 0.90), standardized root mean residual (SRMR, < 0.08), and the root mean square error of approximation (RMSEA; < 0.08). Full information maximum likelihood (FIML) was used to deal with missing data. More specifically, FIML is considered as the state-of-the-art missing data technique when data are considered to be missing at random (Enders, 2010).

For each parameter within the model, standardized regression coefficients were calculated together with a *p*-value. For all parameters, a *p*-value < 0.05 was considered to indicate a statistically significant effect within the model. Significant indirect effects are presented as unstandardized coefficients. In line with suggestions within the literature, we inspected non-symmetric bootstrap *CIs* to assess mediation (Preacher and Hayes, 2008). These intervals were based on 10, 000 bootstrap samples and together provide an empirical representation of the sampling distribution of the indirect effect (*ab*). The indirect effect was considered to be statistically significant if the 95% *CI* did not include zero (Hayes and Rockwood, 2017).

Because the participants were from different schools, we performed sensitivity analyses to investigate if the inclusion of school belonging as a covariate would improve model fit. The Akaike information criterion (AIC) and the Bayesian information criterion (BIC) were inspected to determinate if the inclusion of the covariate improved model fit. The results from all the path analyses showed that the inclusion of school as a covariate did not improve model fit. We therefore decided to present the results for the models without the covariate included.

### Ethics

The study met the intent and requirements of the Health Research Act and the Helsinki Declaration regarding informed consent and unconditional withdrawal and was approved by the Regional Committee for Medical and Health Research Ethics (2016/142). This work was supported by the DAM foundation (2016/FO76521), through the Norwegian Woman’s Public Health Association (H1/2016). A commercial sponsor (TINE AS) supported the study after the study protocol was published (Sundgot-Borgen et al., 2018) but was not involved in data collection, data analysis, or the writing of the present article.

## Results

**FIGURE 2:**
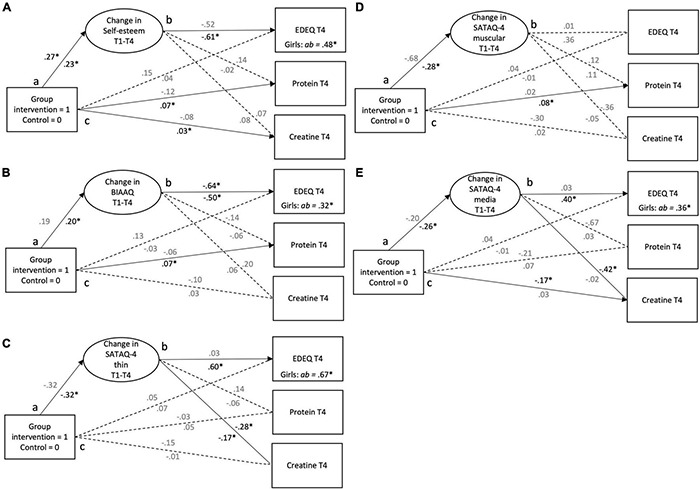
**(A–E)** It presents the direct and indirect effects of the healthy body image (HBI) intervention on eating disorder examination questionnaire (EDEQ), protein supplement use, and creatine supplement use *via* change in **(A)** body image acceptance and action questionnaire (BIAAQ) (body image flexibility), **(B)** self-esteem, **(C)** sociocultural attitudes toward appearance questionnaire-4 (SATAQ-4) thin (thin internalization), **(D)** SATAQ-4 muscular (muscular and athletic internalization), and **(E)** SATAQ-4 media (perceived media pressure). Standardized coefficients for boys are placed above the path and for girls under the path. Indirect effects (ab path) are shown as unstandardized coefficients. Significant coefficients are printed in solid black and non-significant coefficients are printed in gray. T1, baseline; T2, posttest; T3, 3-month follow-up; T4, 12-month follow-up. *p ≤ 0.05. The baseline values for the main outcome variables were included as covariates.

A total of 7,336 data points in the variables of interest had no values and were, therefore, treated as missing data (26.17%). A total of 20,692 data points were included in the analyses. Baseline characteristics and outcomes at T4 in intervention and control boys and girls are shown in Table 2. Control boy dropouts consumed significantly more protein (1.71 vs. 0.97) and creatine (1.50 vs. 0.69) per week than control boy responders, respectively. Control girl dropouts consumed more protein (0.55 *vs.* 0.20) and creatine (0.20 *vs.* 0.04) per week than control girl responders, respectively. No statistically significant differences in psychometric variables at T1 were observed between dropouts and responders. Path analyses and parameter estimates are illustrated in Figures 2A–E and model fit indices and slope estimates are presented in Table 3.

**TABLE 2:** Sample characteristics shown as the mean and SD or percentage and number of observations (N).

	Boys				Girls			
	Intervention		Control		Intervention		Control	
	T1	T4	T1	T4	T1	T4	T1	T4
Sample size (n)	276	155	131	67	590	436	276	177
Age (years)	16.81 (0.46)		16.70 (0.51)		16.79 (0.49)		16.77 (0.50)	
EDEQ	0.96 (1.15)	0.70 (0.90)	1.12 (1.24)	0.69 (0.75)	2.41 (1.61)	1.80 (1.51)	2.66 (1.72)	2.44 (1.83)
Self-esteem	33.01 (5.69)	33.79 (5.99)	32.42 (6.48)	32.68 (6.90)	29.45 (5.95)	30.99 (6.10)	28.47 (6.43)	28.11 (7.42)
BIAAQ	70.53 (9.52)	71.33 (1.60)	70.26 (1.24)	69.77 (1.84)	58.20 (15.71)	62.94 (16.13)	57.00 (16.83)	56.98 (19.39)
SATAQ-4 Thin	2.49 (0.97)	2.07 (0.89)	2.64 (0.92)	2.26 (0.79)	3.31 (1.08)	2.79 (1.13)	3.39 (1.13)	3.28 (1.19)
SATAQ-4 Muscular	3.21 (1.12)	2.77 (1.07)	3.30 (1.03)	3.09 (1.09)	3.02 (1.09)	2.68 (1.03)	2.99 (1.05)	2.74 (1.10)
SATAQ-4 Media	2.10 (1.16)	1.90 (1.03)	2.24 (1.16)	2.06 (1.09)	3.19 (1.24)	2.90 (1.27)	3.22 (1.26)	3.37 (1.23)
Weekly protein use% (n)	22.8 (63)	8.9 (14)	30.7 (40)	29.4 (20)	6.9 (41)	5.0 (22)	7.3 (20)	5.4 (19)
Weekly creatine use% (n)	10.5 (29)	3.1 (5)	17.5 (23)	9.7 (7)	2.3 (14)	1.3 (7)	3.2 (9)	1.8 (3)

*T1, baseline scores; T4, 12-month follow-up scores; BMI, body mass index; EDEQ, ED symptomatology; BIAAQ, body image acceptance and action scale; SATAQ-4 Thin, thinness internalization; SATAQ-4 Muscular, muscular and athletic internalization; SATAQ-4 Media, perceived appearance pressure from media.*

**TABLE 3:** Intercept, slope, and model fit indices for the mediation models for each mediator.

	Self-esteem		BIAAQ		SATAQ-4 thin		SATAQ-4 muscular		SATAQ-4 media	
	Boys	Girls	Boys	Girls	Boys	Girls	Boys	Girls	Boys	Girls
Intercept	32.79^a^	29.23^a^	70.45^a^	58.00^a^	2.49^a^	3.28^a^	3.20^a^	2.93^a^	2.11^a^	3.16^a^
Slope	−0.08^a^	0.30^a^	0.05	0.92^a^	−0.13^a^	−0.11^a^	−0.09^a^	−0.09^a^	−0.05^a^	−0.04^a^
**Model fit indices**										
X2	75.86^b^	123.32^b^	50.29^b^	62.51^b^	66.00^b^	85.21^b^	52.88^b^	99.05^b^	52.13^b^	72.89^b^
CFI	0.92	0.96	0.97	0.99	0.94	0.98	0.96	0.96	0.96	0.97
RMSEA	0.06	0.06	0.04	0.04	0.05	0.05	0.04	0.05	0.04	0.04
90% CI	0.04, 0.07	0.05, 0.07	0.02, 0.05	0.03, 0.05	0.03, 0.06	0.04, 0.06	0.02, 0.05	0.04, 0.06	0.02, 0.06	0.03, 0.05
SRMR	0.12	0.11	0.07	0.03	0.06	0.04	0.05	0.04	0.05	0.03

*Slope, mean change at each timepoint; intercept, mean score at T1.*

*^a^p ≤ 0.05, ^b^p ≤ 0.01.*

*BIAAQ, body image acceptance and action scale; SATAQ-4 Thin, thinness internalization; SATAQ-4 Muscular, muscular and athletic internalization; SATAQ-4 Media, perceived appearance pressure from media.*

### Self-Esteem

As illustrated in Figure 2A, there was a significant positive relationship between group belonging and change in self-esteem indicating a less steep decrease in self-esteem for boys in the intervention group in comparison to boys in the control group (*a* path). In girls, the results showed a significant positive relationship between the group belonging and protein supplement use and creatine supplement use at T4 (*c path).* Also, there was a significant positive relationship between group belonging and change in self-esteem between T1 and T4 (*a* path), as girls in the intervention group showed, on an average, a significant higher increase in self-esteem than the girls in the control group. Also, there was a significant inverse relationship between change in self-esteem and EDEQ at T4 indicating that an increase in self-esteem from T1 to T4 was associated with lower levels of EDEQ at T4 (*b* path). Finally, there was a significant indirect effect of group belonging to EDEQ at T4 *via* change in self-esteem (Figure 2A) in girls.

### Body Image Flexibility

The only significant path for boys was between change in BIAAQ and EDEQ (*b* path) at T4 indicating that an increase in BIAAQ was associated with lower levels of EDEQ (Figure 2A). As shown in Figure 2B, girls in the intervention group used, on an average, significantly more protein supplement use measured at T4 than girls in the control group (*c* path). Girls in the intervention group showed, on an average, a significant higher increase in BIAAQ than the girls in the control group (*a* path). Also, there was a significant inverse relationship between change in BIAAQ and EDEQ at T4 indicating that an increase in BIAAQ was associated with lower levels of EDEQ (*b* path). Last, there was a significant indirect effect of group to EDEQ at T4, *via* change in BIAAQ (*ab* path).

### Thin Internalization

As presented in Figure 2C, in boys, there was a significant inverse relationship between change in SATAQ-4 thin and level of creatine supplement use at T4 (*b* path). For girls, there was a significant inverse relationship between group belonging and change in SATAQ-4 thin between T1 and T4 (*a* path). More specifically, girls in the intervention group showed, on an average, larger decrease in SATAQ-4 thin compared with the girls in the control group. Also, there was a significant positive relationship between change in SATAQ-4 thin and EDEQ at T4, as well as a significant inverse relationship between change in SATAQ-4 thin and creatine supplement use (*b* paths). This indicates that a greater decrease in SATAQ-4 thin from T1 to T4 was associated with lower EDEQ, but more creatine supplement use at T4. Lastly, there was a significant indirect effect of group belonging to EDEQ as well as creatine supplement use both measured at T4, *via* change in SATAQ-4 thin.

### Muscular Internalization

As illustrated in Figure 2D, there were no significant direct or indirect effects in boys. In girls, the results showed a significant positive relationship between the group belonging and creatine supplement use at T4 (*c* path). Also, there was a significant inverse relationship between group belonging and change in SATAQ-4 muscular between T1 and T4 (*a* path). More specifically, girls in the intervention group showed, on an average, larger decrease in SATAQ-4 muscular compared with the girls in the control group.

### Perceived Media Pressure

As shown in Figure 2E, there was a significant inverse relationship between group belonging and creatine supplement use measured at T4 in boys (*c* paths). In girls, there was a significant inverse relationship between group belonging and change in SATAQ-4 media between T1 and T4 (*a* path). More specifically, girls in the intervention group showed, on an average, larger decrease in SATAQ-4 media compared with the girls in the control group. Also, there was a significant positive relationship between change in SATAQ-4 media and EDEQ (*b* path). Lastly, there was a significant indirect effect of group belonging to EDEQ in girls (Figure 2E).

## Discussion

The aim of this study was to investigate potential mediators accounting for the effect of the HBI intervention on ED symptomatology and muscle building supplement use in adolescent boys and girls. We hypothesized that the intervention effect on ED symptomatology and muscle building supplement use was mediated by the change in thin appearance internalization, athletic and muscular internalization, perceived media pressure, self-esteem, and body image flexibility in boys and girls.

Our hypothesis was partially supported as the reduction in ED symptomatology observed at the 12-month follow-up in intervention girls was explained by an increase in self-esteem and body image flexibility, and a reduction in thin internalization and perceived pressure from media. However, the change in the tested mediators did not explain the 12-month follow-up score on ED symptomatology in boys or muscle building supplement use in boys and girls.

### Self-Esteem

We found that the increase in self-esteem in intervention girls relative to control girls from baseline to 12-months follow-up explained the lower ED symptomatology among intervention girls at 12-months follow-up. This is a novel finding and is in contrast to a previous study that did not find that self-esteem mediated the effect of an universal ED prevention program on body image or body esteem (Agam-Bitton et al., 2018). There may be several potential explanations why increased self-esteem may facilitate reductions in ED symptomatology. Studies suggest that body dissatisfaction negatively influences self-esteem (Meland et al., 2021), where self-esteem may act as an important mediator between body dissatisfaction and DE through influencing negative affect (Cruz-Sáez et al., 2020). In contrast, other studies suggest that contingencies of self-esteem related to encouraging love, appearance, and social acceptance may protect against ED development as they predict lower body surveillance and higher body satisfaction (Overstreet and Quinn, 2012). Lastly, high self-esteem may explain lower dietary restraint both through lower body dissatisfaction and by possibly reducing appearance internalization and upward appearance comparison (Rodgers et al., 2020). As such, the increased self-esteem observed in the intervention girls could have caused the reduced ED symptomatology either directly or through influencing other risk or protective factors for ED symptomatology. The HBI intervention aimed to make the students recognize and value their own personal characteristics and what they appreciate in their significant others. This may have enhanced beliefs that their personal characteristics are important and valuable, independent of how they look, which is suggested as an important factor for self-esteem (Overstreet and Quinn, 2012). In addition, targeting social media usage and reducing exposure to content that negatively influences body image may have facilitated the increase in self-esteem (Steinsbekk et al., 2021).

### Body Image Flexibility

We also observed a greater increase in body image flexibility over the study period among intervention girls compared with control girls. This intervention effect on body image flexibility further explains why intervention girls, relative to control girls, had reduced their ED symptomatology 12-months after the intervention. Body image flexibility is an important component of positive body image, and a measure of the ability to openly experience and constructively cope with emotions, events, and thoughts challenging their body image (Sandoz et al., 2013). Therefore, an increase in body image flexibility may result in less engagement in unhealthy appearance modifying behavior; a characteristic and less favorable coping strategy to stress. Hence, an increased body image flexibility may protect against developing EDs. Body image flexibility is associated with numerous psychological outcomes related to well-being and psychopathology (Linardon et al., 2021). However, most studies are cross-sectional and do not investigate the causal relationship. Therefore, our finding, that the change in body image flexibility over 12-months predicted the 12-month intervention effect on ED symptomatology in regular adolescent girls, is therefore a novel and encouraging finding that is not described elsewhere. The previous research finds that holding a more positive body image is associated with higher self-care, being more connected to one’s body, and being more engaged in health promotive behaviors (Homan and Tylka, 2014; Piran, 2015, 2019). Promoting positive body image and embodiment was targeted in the HBI intervention by challenging the students to recognize how lifestyle choices made them feel. They were encouraged to intentionally make lifestyle choices that could enhance their own body experience and functionality rather than focusing on appearance. In addition, the content aiming to make the students recognize unhealthy and untrustworthy lifestyle information from influencers and media in general could have made them more literate against the use of unhealthy methods to change appearance, and rather stay true to their own chosen values reflected in strengthened body image flexibility.

Our findings, that both body image flexibility and self-esteem act as significant mediators for the intervention effect on ED symptomatology, support the suggestion that a health promotive approach with a focus on positive body image may be beneficial in future programs aiming to prevent EDs in girls from an universal population (Piran, 2015).

### Perceived Appearance Pressure and Internalization

The mediating effect of perceived appearance pressure and thin appearance internalization, is in line with different sociocultural and biopsychosocial models of EDs (Rodgers et al., 2014, 2015; Girard et al., 2018b). These models suggest that perceive appearance pressure explains DE or ED symptomatology through the pathways of increased appearance internalization, appearance comparison, and negative body image. This means that the reduction in perceived appearance pressure is expected to make girls less likely to compare themselves to unrealistic appearance standards, which in turn reduces appearance internalization (Rodgers et al., 2015; Stice and van Ryzin, 2019). The favorable changes in perceived appearance pressure and thin appearance internalization observed among our intervention girls could, in light of sociocultural models, have resulted in the girls being less concerned about their appearance, prevented ED symptomatology, and finally reduced their risk of developing EDs (Rodgers et al., 2015, 2020; Stice and van Ryzin, 2019). Several pathways to reduce appearance internalization and its negative impact on body image and ED risk have been suggested in the literature. McLean et al. (2013, 2016) describe the importance of increased media literacy to strengthen critical thinking and evaluation of media messages related to appearance. This is thought to reduce internalization and it’s negative impact on body image and ED risk (McLean et al., 2013, 2016). The HBI intervention targeted media literacy (i.e., the typical idealization portrayed in social media, the origin of idealization, and personal reflection on emotional responses), which may have strengthened the girls media literacy and made them more resilient toward social media messages and thin appearance internalization. The students were also challenged to be more selective on what social media content they would prioritize, in order to feel good about themselves. Addressing the perfectionistic life in social media, and creating an arena for the students to reflect and discuss on this, may have made the girls more aware, critical, and literate (Jeong et al., 2012). The intervention content addressing the perfectionistic lifestyles could also have strengthened the girl’s health literacy, which may be a successful strategy when aiming to improve body image, reduce appearance internalization, and reduce the risk for ED development (Zuair and Sopory, 2020). The result from such discussions and reflections could be a development of cognitive dissonance against idealized appearance standards (Stice et al., 2015). Lastly, Wade et al. (2017) point out the importance of change in attitudes toward appearance standards in the peer environment in ED prevention. Positive change in the peer environment was also aimed for within the HBI intervention and could have acted as another pathway toward reduced appearance pressure and internalization in our sample. Peers plays an important role in adolescents body image and DE as they may be important sources of appearance pressure (Kenny et al., 2017) and predict body dissatisfaction and DE in adolescents (Amaya-Hernández et al., 2017). The HBI intervention aimed to create a collective change in attitudes toward appearance by discussing and providing the students tools and skills to create a more body image friendly and accepting peer environment with less emphasis on appearance. To reduce unhealthy appearance focus transmitting in the peer environment, the HBI intervention aimed to reduce “fat talk” and worked with the students to eliminate appearance focus in their daily communication, and practice focusing on personal characteristics in their communication with their peers. In addition, the HBI intervention aimed to make the students aware of how they could be positive influences for their friends and peers in social media and make them less likely to “like,” “share” comment and post appearance focused content on different social media platforms. Additionally, a novel finding was that targeting and creating change in muscular appearance internalization did not explain the girls ED symptomatology 12-months after the intervention. This suggests that targeting muscular idealization may be unnecessary in future ED prevention interventions in girls.

However, other factors may also explain the relationship among the intervention, the tested mediators, and ED symptomatology 12-months after the intervention. The intervention girls scored higher on self-esteem and lower on ED symptomatology than the control girls at baseline. This may indicate that girls from the intervention group already possessed several favorable resources making them more robust toward challenging life events and emotions. These resources might also make these girls more adaptable to further development of self-esteem, body image flexibility, appearance internalization, perceived media pressure, and ED symptomatology (De Ruiter et al., 2017; Gurung et al., 2019).

None of the tested mediators accounted for ED symptomatology at the 12-month follow-up in boys. This may be explained by the fact that we did not observe any effect of the HBI intervention on ED symptomatology in boys in the effect analyses (Svantorp-Tveiten et al., 2021b). A direct effect between group and body image flexibility, and between body image flexibility and ED symptomatology, was observed. This suggests that strengthening body image flexibility could prevent ED symptomatology in boys. Our results, however, also indicate that the HBI intervention did not create enough enhancements in body image flexibility to create any reductions in ED symptomatology among boys. Surprisingly, our results also suggest that creating enhancements in self-esteem, reduced perceived media pressure, and appearance internalization not necessarily facilitates changes in ED symptomatology in boys as no other direct effects between the mediator and ED symptomatology were observed.

### Muscle Building Supplement Use

We have previously found that the HBI intervention had no effect on muscle building supplement use in girls (Svantorp-Tveiten et al., 2021b). However, we assume that the association found between muscle building supplement use and dropout at follow-up in boys and girls most likely has influenced the results and ability to draw conclusions. Possibly influenced by dropout, the mediation analyses showed more frequent supplement use in intervention girls relative to control girls at 12-month follow-up, which was partly explained by a reduction in thin internalization. This is in contrast to previous research findings in girls that muscle building behaviors or muscularity concerns are positively explained by appearance pressure and appearance internalization either directly (Girard et al., 2018b) or through the pathway of appearance comparison and body dissatisfaction (Rodgers et al., 2020). As such, our results highlight a possible complexity when aiming to prevent *both* muscle building supplement use *and* ED development in girls, which has not been described previously. Similar findings were present in boys as our results suggest that a greater reduction in perceived appearance pressure and thin internalization explained more creatine supplement use in boys at 12-months follow-up independent of group belonging. These findings are in contrast to previous literature explaining muscle building behaviors that suggest that higher appearance pressure and muscular appearance internalization may lead to muscle building behaviors (Girard et al., 2018a; Rodgers et al., 2020). Our finding may indicate that the use of muscle building supplements is multifaceted in nature and that adolescents use muscle building supplements for other reasons than enhancing muscularity. Especially in girls, where we previously found that use was not associated with ED risk factors, and that engagement in sport and exercise were important independent explanatory factors for muscle building supplement use in boys (Svantorp-Tveiten et al., 2021a).

Even if the HBI intervention prevented muscle building supplement use in boys (Svantorp-Tveiten et al., 2021b), none of the tested mediators accounted for the prevention effect on muscle building supplement use in boys. These findings indicate that enhancing self-esteem and body image flexibility may be unnecessary if the only aim is to prevent muscle building supplement use in boys. It may also suggest that other topics in the HBI intervention, which were not measured, were the reason for these changes, such as increased knowledge and literacy toward nutrition, exercise, and health information (i.e., eating well-balanced, regular meals with ordinary food is more important that using supplements). Our results on muscle building supplement use are in line with the findings from a roughly comparable study aiming to prevent muscle building supplement use and doping (Lucidi et al., 2017). This study found that using a media literacy approach and targeting nutritional knowledge and literacy prevented the use of muscle building supplements. The hypothesis that increased knowledge may explain the effect in supplement use among boys may be strengthened by previous mediation analyses from the ATHENA program showing that improving knowledge about nutrition and doping was the initial mediator accounting for the change in several psychological and attitudinal constructs explaining doping intentions and behaviors (Ranby et al., 2009).

### Strengths and Limitations

One of the strengths of this study was the use of a cluster randomized control trial with a longitudinal design including four measurement timepoints allowing for testing change over time and use of standardized and validated psychological instruments. Another strength is the inclusion of several potential mediators, analyses stratified by gender, and including mediators not tested previously in universal ED prevention interventions, and the fact that this is the first study to investigate mediating factors for intervention effects on muscle building supplement use in a universal sample of boys and girls.

Moreover, the sample size was larger than in most previous prevention studies. However, we faced a substantial loss to follow-up and reduced statistical power at the 12-month follow-up. Also, dropout status was associated with muscle building supplement use in the control group. The randomization was performed at a school level to limit the risk of spillover effects between intervention and control students. However, randomization on school level may have increased the risk for self-selection bias and a possibly healthier intervention group compared with control group at baseline (e.g., individuals with more positive outcomes chose to participate in the HBI intervention).

In addition, the use of single mediation models limits the ability to draw conclusion about how interactions between the different mediators might have influenced the outcomes. Moreover, the analyses did not allow for randomization of the relationship between the change in mediator and the outcome. Therefore, caution should be made when evaluating the casual relationship between the estimated mediator slope and the main outcome. The relationship between the mediator and the outcome is contemporary as the change in the mediator and the outcome occurs within the same time period (Zhu et al., 2021). The change in outcome may, however, causes the change in the mediator, when the mediator and the outcome are measured at the same time (Holland, 1988).

### Implications

The professionals working with adolescent girls in an universal setting could aim to increase the girls’ health and media literacy, to stimulate reflections about possible intentions and impacts of (social) media messages, and to strengthen their resilience toward unhealthy and unrealistic appearance standards, notably a thin appearance ideal. Also, the professionals could have additional focus on enhancing the adolescents’ ability to accept and constructively cope with thought, emotions, and experiences challenging their body image, and perform tasks and discussions to strengthen the adolescent girls’ self-esteem. Future studies should investigate the casual relationship between psychological and behavioral factors and muscle building supplement use in both girls and boys, as our study did not confirm previous research explaining muscle building behaviors. In addition, more knowledge on the mechanisms of change related to ED symptomatology in boys are warranted. Future studies could also investigate mediators in a serial multiple mediation model to disentangle how the potential mediators are related and how they together explain changes in ED symptomatology and muscle building supplement use in boys and girls.

## Conclusion

The results support that the HBI intervention is successful in changing mediators within the sociocultural theoretical model of EDs and positive body image in girls. Our results suggest that targeting self-esteem, body image flexibility, perceived appearance pressure, and thin appearance internalization are beneficial when aiming to reduce the risk for ED development in adolescent girls, but not in boys. None of the suggested mediators acted as mediators for muscle building supplement use in neither boys nor girls.

## Data Availability Statement

The raw data supporting the conclusions of this article will be made available by the authors, without undue reservation.

## Ethics Statement

The studies involving human participants were reviewed and approved by the Regional Committee for Medical and Health Research Ethics (2016/142). Written informed consent from the participants’ legal guardian/next of kin was not required to participate in this study in accordance with the national legislation and the institutional requirements.

## Author Contributions

KS-T, OF, MT, CS-B, SB-S, JR, GP, and JS-B contributed to the design and implementation of the research. AI, KS-T, and JS-B contributed to the analysis of the results. All authors contributed to the article and approved the submitted version.

## Conflict of Interest

The authors declare that the research was conducted in the absence of any commercial or financial relationships that could be construed as a potential conflict of interest.

## Publisher’s Note

All claims expressed in this article are solely those of the authors and do not necessarily represent those of their affiliated organizations, or those of the publisher, the editors and the reviewers. Any product that may be evaluated in this article, or claim that may be made by its manufacturer, is not guaranteed or endorsed by the publisher.

## References

[B1] Agam-Bitton R. Abu Ahmad W. Golan M. (2018). Girls-only vs. mixed-gender groups in the delivery of a universal wellness programme among adolescents: A cluster-randomized controlled trial. *PLoS One* 13:e0198872. 10.1371/journal.pone.0198872 29912918 PMC6005464

[B2] Amaya-Hernández A. Alvarez-Rayón G. Ortega-Luyando M. Mancilla-Díaz J. M. (2017). Peer influence in preadolescents and adolescents: A predictor of body dissatisfaction and disordered eating behaviors. *Rev. Mex. Trast. Aliment.* 8 31–39. 10.1016/j.rmta.2016.12.001

[B3] Burke N. L. Schaefer L. M. Karvay Y. G. Bardone-Cone A. M. Frederick D. A. Schaumberg K. (2021). Does the tripartite influence model of body image and eating pathology function similarly across racial/ethnic groups of White, Black, Latina, and Asian women? *Eat. Behav.* 42:101519. 10.1016/j.eatbeh.2021.101519 34022625

[B4] Carvalho P. H. B. Ferreira M. E. C. (2020). Disordered eating and body change behaviours: support for the Tripartite Influence Model among Brazilian male university students. *Ciência Saúde Coletiva* 25 4485–4495. 10.1590/1413-812320202511.35572018 33175056

[B5] Cruz-Sáez S. Pascual A. Wlodarczyk A. Echeburúa E. (2020). The effect of body dissatisfaction on disordered eating: The mediating role of self-esteem and negative affect in male and female adolescents. *J. Health Psychol.* 25 1098–1108. 10.1177/1359105317748734 30101609

[B6] De Ruiter N. M. Van Geert P. L. Kunnen E. S. (2017). Explaining the “how” of self-esteem development: The self-organizing self-esteem model. *Rev. General Psychol.* 21 49–68.

[B7] Doley J. R. McLean S. A. Griffiths S. Yager Z. (2021). Designing body image and eating disorder prevention programs for boys and men: Theoretical, practical, and logistical considerations from boys, parents, teachers, and experts. *Psychol. Men Mascul.* 22 124.

[B8] Enders C. K. (2010). *Applied Missing Data Analysis.* New York, NY: Guilford press.

[B9] Fairburn C. G. Beglin S. J. (2008). Eating disorder examination questionnaire. *Cogn. Behav. Therapy Eat. Disor.* 309:313.

[B10] Friborg O. Reas D. L. Rosenvinge J. H. Ro O. (2013). Core pathology of eating disorders as measured by the Eating Disorder Examination Questionnaire (EDE-Q): the predictive role of a nested general (g) and primary factors. *Int. J. Methods Psych. Res.* 22 195–203. 10.1002/mpr.1389 24038315 PMC6878513

[B11] Girard M. Chabrol H. Rodgers R. F. (2018a). Support for a Modified Tripartite Dual Pathway Model of Body Image Concerns and Risky Body Change Behaviors in French Young Men. *Sex Roles* 78 799–809. 10.1007/s11199-017-0850-z

[B12] Girard M. Rodgers R. F. Chabrol H. (2018b). Prospective predictors of body dissatisfaction, drive for thinness, and muscularity concerns among young women in France: A sociocultural model. *Body Image* 26 103–110. 10.1016/j.bodyim.2018.07.001 30041070

[B13] Gumz A. Weigel A. Daubmann A. Wegscheider K. Romer G. Lowe B. (2017). Efficacy of a prevention program for eating disorders in schools: a cluster-randomized controlled trial. *BMC Psych.* 17:293. 10.1186/s12888-017-1454-4 28800753 PMC5553667

[B14] Gurung U. N. Sampath H. Soohinda G. Dutta S. (2019). Self-esteem as a protective factor against adolescent psychopathology in the face of stressful life events. *J. Indian Assoc. Child Adolesc. Mental Health* 15 34–54.

[B15] Hayes A. F. Rockwood N. J. (2017). Regression-based statistical mediation and moderation analysis in clinical research: Observations, recommendations, and implementation. *Behav. Res. Therapy* 98 39–57. 10.1016/j.brat.2016.11.001 27865431

[B16] Holland P. W. (1988). Causal inference, path analysis and recursive structural equations models. *ETS Res. Rep. Ser.* 1988 i–50. 10.1186/s12711-021-00611-8 33593272 PMC7885440

[B17] Homan K. J. Tylka T. L. (2014). Appearance-based exercise motivation moderates the relationship between exercise frequency and positive body image. *Body Image* 11 101–108. 10.1016/j.bodyim.2014.01.003 24529336

[B18] Jarman H. K. Marques M. D. McLean S. A. Slater A. Paxton S. J. (2021). Social media, body satisfaction and well-being among adolescents: A mediation model of appearance-ideal internalization and comparison. *Body Image* 36 139–148. 10.1016/j.bodyim.2020.11.005 33285385

[B19] Jeong S.-H. Cho H. Hwang Y. (2012). Media Literacy Interventions: A Meta-Analytic Review. *J. Commun.* 62 454–472. 10.1111/j.1460-2466.2012.01643.x 22736807 PMC3377317

[B20] Jose P. E. (2016). The Merits of Using Longitudinal Mediation. *Educ. Psychol.* 51 331–341. 10.1080/00461520.2016.1207175

[B21] Kenny U. O’Malley-Keighran M.-P. Molcho M. Kelly C. (2017). Peer Influences on Adolescent Body Image: Friends or Foes? *J. Adolesc. Res.* 32 768–799. 10.1177/0743558416665478

[B22] Lantolf J. P. (2000). *Introducing Sociocultural Theory* (Vol. 1). Oxford, UK: Oxford University Press.

[B23] Le L. K. Barendregt J. J. Hay P. Mihalopoulos C. (2017). Prevention of eating disorders: A systematic review and meta-analysis. *Clin. Psychol. Rev*. 53 46–58. 10.1016/j.cpr.2017.02.001 28214633

[B24] Levine M. P. Smolak L. (2016). The role of protective factors in the prevention of negative body image and disordered eating. *Eat. Disor.* 24 39–46. 10.1080/10640266.2015.1113826 26643272

[B25] Linardon J. Anderson C. Messer M. Rodgers R. F. Fuller-Tyszkiewicz M. (2021). Body image flexibility and its correlates: A meta-analysis. *Body Image* 37 188–203. 10.1016/j.bodyim.2021.02.005 33684721

[B26] Lucidi F. Mallia L. Alivernini F. Chirico A. Manganelli S. Galli F. (2017). The Effectiveness of a New School-Based Media Literacy Intervention on Adolescents’ Doping Attitudes and Supplements Use [Original Research]. *Front. Psychol.* 8:749. 10.3389/fpsyg.2017.00749 28536552 PMC5422551

[B27] Maher A. L. Lane B. R. Mulgrew K. E. (2021). Self-compassion and body dissatisfaction in men: Extension of the tripartite influence model. *Psychol. Men Mascul.* 22 345–353. 10.1037/men0000271

[B28] Marsh H. W. (2007). “Application of confirmatory factor analysis and structural equation modeling in sport and exercise psychology,” in *Handbook of Sport Psychology*, eds Tenenbaum G. Eklund R. C. (New Jersey, NJ: John Wiley & Sons, Inc), 774–798. 10.1123/jsep.2014-0330

[B29] McLean S. A. Paxton S. J. Wertheim E. H. (2013). Mediators of the relationship between media literacy and body dissatisfaction in early adolescent girls: Implications for prevention. *Body Image* 10 282–289. 10.1016/j.bodyim.2013.01.009 23465878

[B30] McLean S. A. Paxton S. J. Wertheim E. H. (2016). Does Media Literacy Mitigate Risk for Reduced Body Satisfaction Following Exposure to Thin-Ideal Media? *J. Youth Adolesc.* 45 1678–1695. 10.1007/s10964-016-0440-3 26880285

[B31] Meland E. Breidablik H. J. Thuen F. Samdal G. B. (2021). How body concerns, body mass, self-rated health and self-esteem are mutually impacted in early adolescence: a longitudinal cohort study. *BMC Public Health* 21:496. 10.1186/s12889-021-10553-x 33711967 PMC7953559

[B32] Muthén L. K. Muthén B. O. (2017). *Mplus: Statistical Analysis with Latent Variables: User’s Guide. In (Version 8).*

[B33] Nagata J. M. Peebles R. Hill K. B. Gorrell S. Carlson J. L. (2020). Associations between ergogenic supplement use and eating behaviors among university students. *Eat. Disor.* 29 599–615. 10.1080/10640266.2020.1712637 32129729 PMC7483647

[B34] Overstreet N. M. Quinn D. M. (2012). Contingencies of Self-Worth and Appearance Concerns:Do Domains of Self-Worth Matter? *Psychol. Women Q.* 36 314–325. 10.1177/0361684311435221

[B35] Perey I. Koenigstorfer J. (2020). Appearance Comparisons and Eating Pathology: A Moderated Serial Mediation Analysis Exploring Body Image Flexibility and Body Appreciation as Mediators and Self-Compassion as Moderator. *Body Image* 35 255–264. 10.1016/j.bodyim.2020.09.008 33157399

[B36] Piran N. (2015). New possibilities in the prevention of eating disorders: The introduction of positive body image measures. *Body Image* 14 146–157. 10.1016/j.bodyim.2015.03.008 25886711

[B37] Piran N. (2019). “The experience of embodiment construct: Reflecting the quality of embodied lives,” in *Handbook of Positive Body Image and Embodiment*, eds Tylka T. L. Piran N. (Oxford, UK: Oxford University Press), 11–21. 10.1016/0890-4065(94)90019-1

[B38] Preacher K. J. Hayes A. F. (2008). Asymptotic and resampling strategies for assessing and comparing indirect effects in multiple mediator models. *Behav. Res. Methods* 40 879–891. 10.3758/brm.40.3.879 18697684

[B39] Ranby K. W. Aiken L. S. Mackinnon D. P. Elliot D. L. Moe E. L. McGinnis W. (2009). A mediation analysis of the ATHENA intervention for female athletes: prevention of athletic-enhancing substance use and unhealthy weight loss behaviors. *J. Pediatr. Psychol.* 34 1069–1083. 10.1093/jpepsy/jsp025 19386771 PMC2782253

[B40] Rø Ø Reas D. L. Stedal K. (2015). Eating disorder examination questionnaire (EDE-Q) in Norwegian adults: Discrimination between female controls and eating disorder patients. *Eur. Eat. Disor. Rev.* 23 408–412. 10.1002/erv.2372 26094887

[B41] Rodgers R. F. Chabrol H. Paxton S. J. (2011). An exploration of the tripartite influence model of body dissatisfaction and disordered eating among Australian and French college women. *Body Image* 8 208–215. 10.1016/j.bodyim.2011.04.009 21664887

[B42] Rodgers R. F. Ganchou C. Franko D. L. Chabrol H. (2012). Drive for muscularity and disordered eating among French adolescent boys: A sociocultural model. *Body Image* 9 318–323. 10.1016/j.bodyim.2012.03.002 22494958

[B43] Rodgers R. F. McLean S. A. Paxton S. J. (2015). Longitudinal relationships among internalization of the media ideal, peer social comparison, and body dissatisfaction: Implications for the tripartite influence model. *Devel. Psychol.* 51:706. 10.1037/dev0000013 25751099

[B44] Rodgers R. F. Paxton S. J. McLean S. A. (2014). A biopsychosocial model of body image concerns and disordered eating in early adolescent girls. *J. Youth Adolesc.* 43 814–823. 10.1007/s10964-013-0013-7 24014348

[B45] Rodgers R. F. Slater A. Gordon C. S. McLean S. A. Jarman H. K. Paxton S. J. (2020). A biopsychosocial model of social media use and body image concerns, disordered eating, and muscle-building behaviors among adolescent girls and boys. *J. Youth Adolesc.* 49 399–409. 10.1007/s10964-019-01190-0.pdf 31907699

[B46] Rogers C. B. Webb J. B. Jafari N. (2018). A systematic review of the roles of body image flexibility as correlate, moderator, mediator, and in intervention science (2011–2018). *Body Image* 27 43–60. 10.1016/j.bodyim.2018.08.003 30144730

[B47] Rosenberg M. (1965). *Society and the Adolescent Self-Image.* New Jersey, NJ: Princeton University Press.

[B48] Sandoz E. K. Wilson K. G. Merwin R. M. Kate Kellum K. (2013). Assessment of body image flexibility: The Body Image-Acceptance and Action Questionnaire. *J. Context. Behav. Sci.* 2 39–48. 10.1016/j.jcbs.2013.03.002

[B49] Schaefer L. M. Burke N. L. Thompson J. K. Dedrick R. F. Heinberg L. J. Calogero R. M. (2015). Development and validation of the Sociocultural Attitudes Towards Appearance Questionnaire-4 (SATAQ-4). *Psychol. Assess.* 27 54–67. 10.1037/a0037917 25285718

[B50] Schaefer L. M. Rodgers R. F. Thompson J. K. Griffiths S. (2021). A test of the tripartite influence model of disordered eating among men. *Body Image* 36 172–179. 10.1016/j.bodyim.2020.11.009 33307310 PMC7987734

[B51] Schwartz C. Drexl K. Fischer A. Fumi M. Löwe B. Naab S. (2019). Universal prevention in eating disorders: A systematic narrative review of recent studies. *Mental Health Prevent.* 14:200162. 10.1002/eat.23242 32048752

[B52] Steinsbekk S. Wichstrøm L. Stenseng F. Nesi J. Hygen B. W. Skalická V. (2021). The impact of social media use on appearance self-esteem from childhood to adolescence – A 3-wave community study. *Comput. Hum. Behav.* 114:106528. 10.1016/j.chb.2020.106528

[B53] Stice E. Becker C. B. Yokum S. (2013). Eating disorder prevention: Current evidence-base and future directions. *Int. J. Eat. Disor.* 46 478–485. 10.1002/eat.22105 23658095 PMC3926692

[B54] Stice E. Shaw H. Becker C. B. Rohde P. (2008). Dissonance-based Interventions for the prevention of eating disorders: using persuasion principles to promote health. *Prevent. Sci.* 9 114–128. 10.1007/s11121-008-0093-x 18506621 PMC2577371

[B55] Stice E. van Ryzin M. J. (2019). A prospective test of the temporal sequencing of risk factor emergence in the dual pathway model of eating disorders. *J. Abnorm. Psychol.* 128:119. 10.1037/abn0000400 30570269 PMC6361717

[B56] Stice E. Yokum S. Waters A. (2015). Dissonance-Based Eating Disorder Prevention Program Reduces Reward Region Response to Thin Models; How Actions Shape Valuation. *PLoS One* 10:e0144530. 10.1371/journal.pone.0144530 26641854 PMC4671712

[B57] Sundgot-Borgen C. Bratland-Sanda S. Engen K. M. E. Pettersen G. Friborg O. Torstveit M. K. (2018). The Norwegian healthy body image programme: study protocol for a randomized controlled school-based intervention to promote positive body image and prevent disordered eating among Norwegian high school students [journal article]. *BMC Psychol.* 6:8. 10.1186/s40359-018-0221-8 29510762 PMC5840729

[B58] Sundgot-Borgen C. Friborg O. Kolle E. Engen K. M. Sundgot-Borgen J. Rosenvinge J. H. (2019). The healthy body image (HBI) intervention: Effects of a school-based cluster-randomized controlled trial with 12-months follow-up. *Body Image* 29 122–131. 10.1016/j.bodyim.2019.03.007 30928681

[B59] Sundgot-Borgen C. Friborg O. Kolle E. Torstveit M. K. Sundgot-Borgen J. Engen K. M. E. (2020a). Does the Healthy Body Image program improve lifestyle habits among high school students? A randomized controlled trial with 12-month follow-up. *J. Int. Med. Res.* 48:0300060519889453. 10.1177/0300060519889453 31802697 PMC7607281

[B60] Sundgot-Borgen C. Stenling A. Rosenvinge J. H. Pettersen G. Friborg O. Sundgot-Borgen J. (2020b). The Norwegian Healthy Body Image Intervention promotes Positive Embodiment Through Improved Self-Esteem. *Body Image* 35 84–95. 10.1016/j.bodyim.2020.08.014 33022483

[B61] Svantorp-Tveiten K. M. E. Friborg O. Torstveit M. K. Mathisen T. F. Sundgot-Borgen C. Rosenvinge J. H. (2021a). Protein, creatine and dieting supplements among adolescents: Use and associations with eating disorder risk factors, exercise- and sports participation, and immigrant status. *Front. Sports Active Living* 3:372. 10.3389/fspor.2021.727372 34723179 PMC8548763

[B62] Svantorp-Tveiten K. M. E. Torstveit M. K. Rosenvinge J. H. Sundgot-Borgen C. Friborg O. Bratland-Sanda S. (2021b). Effect of A Healthy Body Image intervention on risk- and protective factors for eating disorders: A cluster randomized controlled trial. *Mental Health Prevent.* 24:200225.

[B63] Tylka T. L. Wood-Barcalow N. L. (2015). What is and what is not positive body image? Conceptual foundations and construct definition. *Body Image* 14 118–129. 10.1016/j.bodyim.2015.04.001 25921657

[B64] Vickers A. J. Altman D. G. (2001). Analysing controlled trials with baseline and follow up measurements. *BMJ* 323 1123–1124. 11701584 10.1136/bmj.323.7321.1123 PMC1121605

[B65] Wade T. D. Wilksch S. M. Paxton S. J. Byrne S. M. Austin S. B. (2017). Do universal media literacy programs have an effect on weight and shape concern by influencing media internalization? *Int. J. Eat. Disord.* 50 731–738. 10.1002/eat.22689 28152232

[B66] Watson H. J. Joyce T. French E. Willan V. Kane R. T. Tanner-Smith E. E. (2016). Prevention of eating disorders: A systematic review of randomized, controlled trials. *Int. J. Eat. Disord.* 49 833–862. 10.1002/eat.22577 27425572

[B67] Webb J. B. (2015). Body image flexibility contributes to explaining the link between body dissatisfaction and body appreciation in White college-bound females. *J. Context. Behav. Sci.* 4 176–183. 10.1016/j.jcbs.2015.06.001

[B68] Yager Z. Diedrichs P. C. Ricciardelli L. A. Halliwell E. (2013). What works in secondary schools? A systematic review of classroom-based body image programs. *Body Image* 10 271–281. 10.1016/j.bodyim.2013.04.001 23683611

[B69] Yager Z. McLean S. A. (2020). Muscle building supplement use in Australian adolescent boys: relationships with body image, weight lifting, and sports engagement. *BMC Pediatr.* 20:89. 10.1186/s12887-020-1993-6 32101154 PMC7043030

[B70] Yager Z. O’Dea J. A. (2014). Relationships between body image, nutritional supplement use, and attitudes towards doping in sport among adolescent boys: implications for prevention programs. *J. Int. Soc. Sports Nutr.* 11 13. 10.1186/1550-2783-11-13 24670105 PMC3986904

[B71] Zhu S. Sagherian K. Wang Y. Nahm E.-S. Friedmann E. (2021). Longitudinal Moderated Mediation Analysis in Parallel Process Latent Growth Curve Modeling in Intervention Studies. *Nurs. Res.* 70 184–192. 10.1097/nnr.0000000000000503 33528237

[B72] Zuair A. A. Sopory P. (2020). Effects of media health literacy school-based interventions on adolescents’ body image concerns, eating concerns, and thin-internalization attitudes: A systematic review and meta-analysis. *Health Commun.* 37 20–28. 10.1080/10410236.2020.1813954 32873082

